# Cucurbitacin B Induces Hypoglycemic Effect in Diabetic Mice by Regulation of AMP-Activated Protein Kinase Alpha and Glucagon-Like Peptide-1 via Bitter Taste Receptor Signaling

**DOI:** 10.3389/fphar.2018.01071

**Published:** 2018-09-21

**Authors:** Kang-Hoon Kim, In-Seung Lee, Ji Young Park, Yumi Kim, Eun-Jin An, Hyeung-Jin Jang

**Affiliations:** ^1^Department of Science in Korean Medicine, Graduate School, Kyung Hee University, Seoul, South Korea; ^2^Department of Biochemistry, Graduate School, Kyung Hee University, Seoul, South Korea

**Keywords:** cucurbitacin B, taste receptor, GLP-1, AMPK, diabetes, alpha-gustducin, enteroendocrine L-cells

## Abstract

Taste receptors exist in several organs from tongue to colon and have diverse functions dependent on specific cell type. In enteroendocrine L-cells, stimulation of taste receptor signaling induces incretin hormones. Among incretin hormones, glucagon-like peptide-1 (GLP-1) induces insulinotropic action by activating GLP-1 receptor of pancreatic β-cells. However, GLP-1 mimetic medicines have reported clinical side effects, such as autoimmune hepatitis, acute kidney injury, pancreatitis, and pancreatic cancer. Here, we hypothesized that if natural components in ethnomedicines can activate agonistic action of taste receptor; they may stimulate GLP-1 and therefore, could be developed as safe and applicable medicines to type 2 diabetes mellitus (T2DM) with minimal side effects. Cucurbitacin B (CuB) is composed of triterpenoid structure and its structural character, that represents bitterness, can stimulate AMP-activated protein kinase (AMPK) pathway. CuB ameliorated hyperglycemia by activating intestinal AMPK levels and by inducing plasma GLP-1 and insulin release in diabetic mice. This hypoglycemic action was decreased in dorsomorphin-injected mice and α-gustducin null mice. Moreover, systemic inhibition study in differentiated NCI-H716 cell line showed that CuB-mediated GLP-1 secretion was involved in activation of AMPK through α-gustducin and Gβγ-signaling of taste receptors. In summary, we conclude that, CuB represents novel hypoglycemic agents by activation of AMPK and stimulation of GLP-1 in differentiated enteroendocrine L-cells. These results suggest that taste receptor signaling-based therapeutic agents within tremendously diverse ethnomedicines, could be applied to developing therapeutics for T2DM patients.

## Introduction

Bitter taste instinctively causes aversion, which helps protect humans from ingesting toxic substances (Chandrashekar et al., 2000). Bitter taste receptors, which utilize G-protein-coupled receptors (GPCR) are present from tongue to colon, and their signaling transductions and functional roles under investigation (Jang et al., 2007; Janssen et al., 2011). When bitter chemicals are consumed, the stomach induces a repulsive behavior after detecting bitter taste on brush cells, and the gastrointestinal tract provides a protection mechanism by preventing ingestion (Janssen et al., 2011). Furthermore, in the gut, several bitter compounds and bitter herbals in ethnomedicines stimulate anorexigenic or orexigenic hormones with minimal side effects (Kim and Jang, 2016).

Enteroendocrine cells in the epithelium of the stomach and intestines secrete more than 50 different peptides as functional hormones (Gribble and Reimann, 2016). Among anorexigenic hormones, Glucagon-like peptide-1 (GLP-1) and peptide YY (PYY) are postprandially co-secreted in enteroendocrine L-cells (L-cells) of the gut (Gribble and Reimann, 2016). GLP-1, discovered in 1985 as a second incretin hormone, is secreted through taste receptor signaling in L-cells, and induces insulinotrophic action (Schmidt et al., 1985). For perception of a specific taste by taste receptors, the G-protein α subunit of gustducin (Gα-gust) stimulates Phosphodiesterase (PDE) or G-protein β and γ subunits of gustducin as a second messenger-signaling cascade for the Gα-gust-mediated activation of phospholipase β_2_ (PLCβ_2_) (Margolskee, 2002; Jang et al., 2007). In L-cells, the downstream signaling of taste receptor results in stimulation of neurotransmitters, such as GLP-1 and regulation associated with opening of ion channels, such as transient receptor potential channel 5 (TRPM5) and calcium influx (Kinnamon, 2012).

Several T2DM medicines, that harness GLP-1 action, have been developed in clinical studies. Among them, exenatide, sitagliptin, and liraglutide have shown significant amelioration of diabetic markers such as high hyperglycemia level and hemoglobin A1c level (Kim and Egan, 2008). However, while GLP-1 receptor (GLP-1R) agonistic agents are effective medicines for T2DM, some unavoidable side effects include nausea, vomit, autoimmune hepatitis, acute kidney injury, pancreatitis, and pancreatic cancer (Prasad-Reddy and Isaacs, 2015). The risks of GLP-1R based-therapy, indicate that many T2DM patients will benefit from development of safer medicine.

To reduce the detrimental side effects of GLP-1R based-therapy for T2DM medicines, traditional herbal medicines have been prescribed to prevent hyperglycemia for T2DM patients. Because many ethnomedicines and natural components showed to alleviate hyperglycemia in T2DM patients with minimal side effects (Kim and Jang, 2015, 2016). Especially, CuB including cucurbitane triterpenoid from bitter melon or other phytochemicals, has been reported to activate AMP-activated protein kinase (AMPK) (Tan et al., 2008). AMPK are associated with both acute and chronic effects on glucose and lipid metabolism through the phosphorylation of key protein substrates (Andersson et al., 2004; Kim et al., 2017a). In addition, AMPK, acts as a sensor of cellular energy homeostasis, and regulates metabolic diseases and AMPK signaling at the cellular level (Kahn et al., 2005). In the intestinal L-cells, however, the role of AMPK in regulating energy homeostasis and its functional effects is unknown. In this study, we investigated the functional role of AMPK activation in L-cells. We found that AMPK activation led to secretion of GLP-1, and the signal transduction was mediated by Gα-gust. Our results suggest that a wider variety of taste receptor signaling molecules could be applied for development of novel therapeutic agents, as GLP-1 secretagogues and for patients with metabolic diseases, including obesity and T2DM using CuB. Moreover, ethnopharmacological medicines including cucurbitane triterpenoid structure may provide rich resources to discover novel taste receptor binding natural components for treatment of patients with metabolic disorders with minimal side effects.

## Materials and Methods

### Chemical

Cucurbitacin B (CuB, 100 μM), glucose (10%), denatonium benzoate (10 mM), 2-APB (10 μM), U73122 (10 μM), U73343 (10 μM), rolipram (50 μM), STO-690 (100 μM), Dorsomorphin (50 μM), forskolin (5 μM), and AICAR (5-Aminoimidazole-4-carboxamide-1-β-D-ribofuranoside, 1–5 mM) were purchased from Sigma-Aldrich (Sigma-Aldrich, MO, United States). Gallein (10 μM) was purchased from Santa Cruz Biotechnology (Santa Cruz, CA, United States). Lactisole (10 μM) was purchased from Endeavour Speciality Chemicals (Daventry, United Kingdom). Metformin, Diabex tab was purchased from Daewoong Pharmaceutical Co., Ltd. (Gyeonggi-do, South Korea). PBS was purchased from Corning (NY, United States) Bovine serum albumin (BSA) was purchased from RMBIO (MT, United States). Exendin 9-39 was purchased from Abcam (Cambridge, United Kingdom).

### Culture of NCI-H716 Cells

NCI-H716 cells were maintained in suspension culture as described by the American Type Culture Collection (Manassas, VA, United States). Two days before the experiments, NCI-H716 cells were seeded into 24-well culture plates pre-coated with Matrigel (Reimer et al., 2001). Because, NCI-H716 cells became differentiated, it made the cells express several neuroendocrine markers, such as chromogranin A, and became a qualified endocrine cellular model for conducting the regulation of GLP-1 secretion on Matrigel. Cells were incubated in 5% CO_2_ incubator for 1 h at 37°C with different test agents or with 0.01% DMSO as the vehicle. The culture medium was collected for the measurement of GLP-1 by Bio-Plex^®^ MAGPIX^TM^ Multiplex Reader (Bio-Rad, CA, United States). The results were analyzed with Bio-Plex 1 Manager software (Bio-Rad). All results of GLP-1 study were normalized by quantitative protein of NCI-H716 cells per well. A cell viability assay was performed using 3-(4,5-dimethylthiazol-2-yl)-2,5-diphenyltetrazolium bromide (MTT; Invitrogen, CA, United States) according to the manufacturer’s instructions.

### Real-time PCR of NCI-H716 Cells

The expression of bitter taste receptors in NCI-H716 cells was determined with real-time quantitative PCR as described in the manufacturer’s guide (Life Technologies, NY, United States) and was performed previously as described by Kim et al. (2014a, 2015) and Hong et al. (2018). 2^−ΔΔ*Ct*^ value compared to the Gα-gust (*GNAT3*) was determined with StepOne Software (Life Technologies, NY, United States). *GAPDH* was used as an endogenous control (Phannasorn et al., 2017). The results are from three to five individual experiments performed in triplicate. The information of the primers indicted at **Table 1**.

**Table 1:** Sequence information for real-time quantitative PCR primer.

Gene	Forward	Reverse
*GAPDH*	GCCACATCGCTCAGACACC	CCCAATACGACCAAATCCGT
*GNAT3*	CTATGACATGGTCCTCGTGGAA	GATACTGTTGAACAGGTGAAGGCTT
*T2R10*	CATTTCCCTTTGGAGACACAAC	ATGAGCTTCTGTGTTGGAGTC

### Animals

The original mating pairs of homozygous Gα-gust null mice were kindly provided by Dr. Robert F. Margolskee (Monell Chemical Senses Center, Philadelphia, PA, United States) as described by Jang et al. (2007). Gα-gust null mice of seven-week old male were used in *in vivo* study. The seven-week old male of LEPR−/− (*db/db*) mice and of C57BL/6 were purchased from Daehan Biolink (DBL, Eumseong-gun, Chungcheongbuk-do, South Korea). All mice were acclimated for 1 week in a room with a light-dark cycle of 12 h at a temperature ranging from 21 to 23°C and moderate humidity (55–60%). Food and water were provided *ad libitum*.

### Oral Glucose Tolerance Test

To test oral glucose tolerance test (OGTT, 5 g/kg), mice were incubated for 15 h under fasting condition. The i.v injection of dorsomorphin (2 mg/kg) or vehicle (PBS, 0.01% DMSO) was performed and incubated for 30 min before OGTT (Song et al., 2013). The i.v injection of dorsomorphin (2 mg/kg) performed in C57BL/6 mice for 30 min before the glucose administration (Song et al., 2013). Under OGTT condition, CuB (0.1 mg/kg) orally was administrated in C57BL/6 (*n* = 8) or Gα-gust^−/−^ mice (*n* = 5). The i.p injection of exendin 9-39 (Ex9, 10 μg/100 ul) or vehicle (PBS, 1% BSA) was performed and incubated for 20 min before OGTT (Kim et al., 2017c). The experiments were repeated with the other groups of C57BL/6 mice (*n* = 8). All *in vivo* studies were performed at least twice using the other sets of mice. Blood glucose was measured from the tail vein using the Accu-Chek Performa system (Roche Diagnostics, Mannheim, Germany) at six time points: 0 (before glucose gavage), 10 (10 min after glucose gavage), 20, 40, 90, and 120 min. In *db/db* mice, each mouse group was divided into three groups; basal (saline), CuB (0.1 mg/kg), and Met (300 mg/kg) of *n* = 8 and was orally administered just before glucose gavage (5 g/kg). CuB (0.1 mg/kg per day) orally was administrated for 20 days. In order to measure high concentration of blood glucose level (over 600 mg/dL), we diluted blood with blood and PBS (1:1) and performed to measure blood glucose level.

### Blood Sampling

Mice were performed by OGTT after fasting for 15 h. The collected blood from the tail veins was immediately transferred into EDTA-coated microcentrifuge tubes containing a dipeptidyl peptidase IV inhibitor (EMD Millipore, United States) and a protease inhibitor cocktail (Roche Diagnostics, Switzerland) as described by Kim et al. (2014a, 2015). After each drug and glucose administration, subsequent 40 μl of blood was collected at 0, 10, 20, and 40 min. Collected samples were centrifuged at 1,000 ×*g* for 10 min at 4°C. The supernatant of each sample was dispensed into a fresh tube and stored at −80°C until analysis. The GLP-1 and insulin concentrations in each sample were measured using a Bio-Plex MAGPIX Multiplex reader (Bio-Rad, CA, United States). The results were analyzed with Bio-Plex Manager software (Bio-Rad, CA, United States).

### Western Blot Analysis

The isolated NCI-H716 cells and duodenal tissues were lysed with cell lysis buffer. The isolated intestinal tissues were lysed with tissue lysis buffer (Thermo Fisher, United States) and immunoblotting was performed as described by Kim et al. (2016, 2017b) and Lee et al. (2017). Individual proteins were detected with primary antibodies against the phosphorylated form of AMPK (p-AMPK, T172; 1:1000), total AMPK (t-AMPK; 1:1,000), CAMKKβ (1:1,000), and β-actin (1:1,000). The p-AMPK and AMPK were purchased from Cell Signaling (Danvers, MA, United States). CAMKKβ and β-actin were purchased from Santa Cruz Biotechnologies (CA, United States) (Jang et al., 2017).

### siRNA Preparation and NCI-H716 Cell Transfection

The predesigned siRNA duplexes for *GNAT3* and *T2R10* were purchased from Bioneer (Bioneer Co., Daejeon, South Korea). A scrambled negative control siRNA was also purchased from Santa Cruz. Transfection with siRNA duplexes was performed with Lipofectamine^®^ RNAiMAX Reagent (Invitrogen, CA, United States) as previously described (Kim et al., 2014a).

### Calcium Imaging

NCI-H716 cells were seeded at clear bottom 96-well black plate (Corning, MA, United States). After differentiation, the media was changed with PBS and incubated 30 min with fura-2 AM dye as described previously (Kim et al., 2014a). Levels of intracellular free [Ca^2+^]_i_ were observed by Nikon Eclipse TS 100 fluorescence imaging system (Nikon Instruments Inc., NY, United States), and quantified by InCyt Im2 software (University of Cincinnati, Cincinnati, OH, United States). The number of observed cells were 10 cells per well (Suh et al., 2015).

### cAMP ELISA

Endocrine differentiated NCI-H716 cells were incubated with CuB, denatonium benzoate, or forskolin. The drug-treated cells were collected at 0, 1, 5, 15, and 30 min. Rolipram was pre-treated prior to CuB treatment. The collected cells were lysed using 0.1 M HCl, and the intracellular cAMP was assayed using ELISA (Enzo, United States) according to the manufacturer’s instructions as described by Kim et al. (2014a).

### Ethics Statement

All animal study protocols were approved by the Institutional Animal Care and Use Committee (IACUC) of Kyung Hee University. The approved number of animal experiment was KHUASP(SE)-14-046.

### Statistical Analysis

GraphPad Prism 5 software (GraphPad Software, San Diego, CA, United States) was used for the statistical analysis of the experimental results. The results from the GLP-1, insulin, blood glucose, cAMP and protein expression level using western blot at least three separate experiments performed in quadruplicate. Data represent the Mean ± SEM. The statistical significance of each bar chart was measured using one-way ANOVA with Dunnett’s *post hoc* (for *in vitro* studies) or Tukey’s *post hoc* (for *in vivo* studies). For *in vivo* studies, two-way ANOVA with Tukey’s *post hoc* (to compare more than three groups) or Bonferroni’s *post hoc* (to compare two groups) were performed (Kim et al., 2017c). The *in vivo* experiments were repeated twice. Seven to eight mice per C57BL/6, five mice per Gα-gust^−/−^, and eight mice per LEPR−/− (*db/db*) were used for the *in vivo* studies. The OGTT and the mouse plasma GLP-1 and insulin hormones studies analyzed by two-way ANOVA, followed by Bonferroni *post hoc* tests at individual time points where applicable. *P* < 0.05 was considered significant.

## Results

### CuB Stimulates GLP-1 Release and Alleviates Hyperglycemia in *db/db* Mice

**FIGURE 1:**
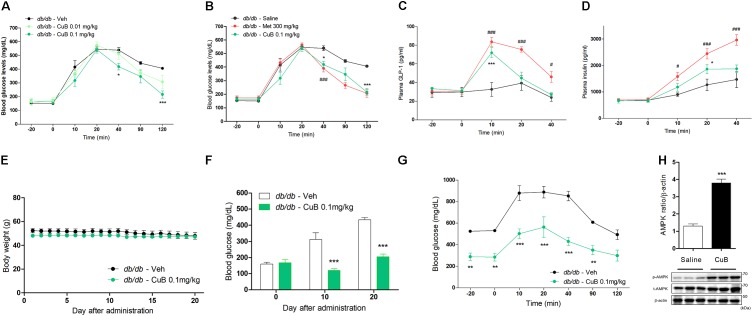
Cucurbitacin B ameliorates hyperglycemia in *db/db* mice. **(A)** Fasted mice were administrated with CuB 0.01 (Mint) and 0.1 mg/kg (Green). **(B)** CuB-mediated hypoglycemic effects were similar to Metformin 300 mg/kg (Red). Blood glucose were measured using Acu-Check performa in end of tail. Data are presented as a Mean ± SEM, *n* = 8 of the LEPR −/− (*db/db*) mice group treated with or without CuB. ^###^*P* < 0.001 (Meformin) vs. vehicle, ^∗∗∗^*P* < 0.001, ^∗^*P* < 0.05 (CuB) vs. vehicle. **(C,D)** The plasma GLP-1 and plasma insulin were measured using multiplex system. Data are presented as a Mean ± SEM, *n* = 8 of the LEPR −/− (*db/db*) mice group treated with or without CuB. ^#^*P* < 0.05, ^###^*P* < 0.001, (Meformin) vs. vehicle ^∗^*P* < 0.05, ^∗∗∗^*P* (CuB) < 0.001 vs. vehicle. Met, Metformin; CuB, Cucurbitacin B. Metformin was used for positive control of hypo-glycaemic agents. **(E)** Body weight was compared with treatment of vehicle or, of CuB 0.1 mg/kg per day and was measured for 20 days on *db/db* mice. Data are presented as a Mean ± SEM, *n* = 8 of the LEPR −/− (*db/db*) mice group treated with or without CuB. **(F)** Every 10 days, fasting blood glucose levels were measured on *db/db* mice. Data are presented as a Mean ± SEM, *n* = 8 of the LEPR −/− (*db/db*) mice group treated with or without CuB. ^∗∗∗^*P* < 0.001 vs. vehicle. **(G)** After treatment with vehicle or, CuB (0.1 mg/kg per day for 20 days), OGTT was performed on *db/db* mice. Data are presented as a Mean ± SEM, *n* = 8 of the LEPR −/− (*db/db*) mice group treated with or without CuB. ^∗∗^*P* < 0.01, ^∗∗∗^*P* < 0.001 (CuB) vs. vehicle. **(H)** The duodenum of protein expression of p-AMPK, and t-AMPK was measured using western blot (a representative blot is shown; *n* = 3). The densitometry of AMPK ratio was shown each other. Data are presented as a Mean ± SEM, *n* = 8. ^∗∗∗^*P* < 0.001 vs. saline.

To evaluate the ameliorative effects of hyperglycemia, we performed an OGTT administrated with CuB in *db/db* mice. The CuB administration (0.1 mg/kg) led to hypoglycemic effects in blood glucose at 40 and 120 min after OGTT (**Figure 1A**). Hypoglycemic effects of CuB administration were similar to Metformin, major hypoglycemia agent (**Figure 1B**). Plasma GLP-1 and insulin levels were increased by CuB administration (**Figures 1C,D**). Moreover, to test whether CuB maintains hypoglycemic conditions in Type 2 diabetes mellitus, we performed CuB administration (0.1 mg/kg) per day for 20 days in *db/db* mice. There were no alterations of body weights (**Figure 1E**). However, we observed greater decreased fasting glucose levels than vehicle group after this CuB administration at days 10 and 20 in *db/db* mice (**Figure 1F**). In addition, remarkable improvement of fasting glucose levels was confirmed through OGTT in *db/db* mice (**Figure 1G**). Moreover, CuB induced duodenal AMPK activation (**Figure 1H**). These results suggest that CuB may attenuate hyperglycemia by regulating fasting glucose levels and stimulating GLP-1 release in diabetic mouse model.

### CuB-Mediated GLP-1 Release Depends on AMPK or Gα-Gust

**FIGURE 2:**
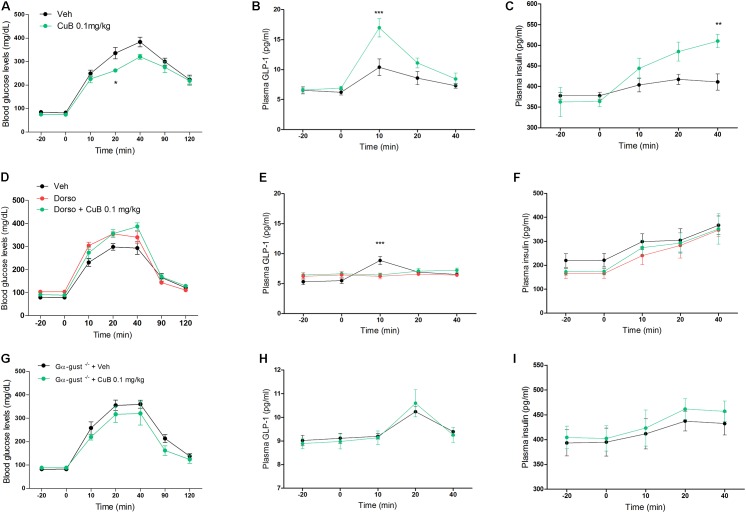
CuB-mediated hypoglycemia is involved in AMPK activation and α-gustducin. **(A)** Fasted mice were administrated with or without CuB 0.1 mg/kg (Green) and with vehicle (Black) before OGTT. **(B,C)** After OGTT, plasma GLP-1 and plasma insulin were measured using multiplex system. Data are presented as a Mean ± SEM, *n* = 8 of the C57bL/6 group treated with or without CuB. ^∗∗^*P* < 0.01, ^∗∗∗^*P* < 0.001 (CuB) vs. vehicle. **(D)** Fasted mice were injected through i.v with dorso (2 mg/kg), with (Green) or without (Red) 0.1 mg/kg CuB before OGTT. **(E,F)** After OGTT, plasma GLP-1 and plasma insulin were measured using multiplex system. Data are presented as a Mean ± SEM, *n* = 7 of the C57BL/6 group treated with or without CuB. ^∗∗∗^*P* < 0.001 (CuB), vs. vehicle. Dorso, Dorsomorphin. **(G)** Fasted mice of Gα-gust^−/−^ mice were administrated with CuB 0.1 mg/kg (Green) and with vehicle (Black) just before OGTT. **(H,I)** After OGTT, plasma GLP-1 and plasma insulin were measured using multiplex system. Data are presented as a Mean ± SEM, *n* = 5 of the male Gα-gust^−/−^ group treated with or without CuB. The blood glucose levels were measured using Acu-Check performa in end of tail.

To examine the GLP-1 secreting effect of CuB corresponding to the *in vitro*, which is of physiological relevance to *in vivo* evidence of diabetic mice upon GLP-1 release, we administered CuB or vehicle to C57BL/6 mice under OGTT. CuB administration showed greater decreases of blood glucose levels (Mean: 263, SD: 7.34) than saline (Mean: 336.14, SD: 24.23) at 20 min (**Figure 2A**). CuB also contributed to the greater increase of plasma GLP-1 release (Mean: 16.96, SD: 1.52) than vehicle (Mean: 10.38, SD: 1.36) at 10 min (**Figure 2B**), and greater induction of plasma insulin release (Mean: 510.13, SD: 16.26) than vehicle (Mean: 411, SD: 19.7) at 40 min (**Figure 2C**). To examine whether CuB-mediated GLP-1 release depended on AMPK activation, we injected with and without dorsomorphin (2 mg/kg). The i.v injected dorsomorphin was shown to eliminate p-AMPK expression levels (**Supplementary Figure 1**). In dorsomorphin-injected mice, the CuB-mediated glucose-lowering effect and GLP-1 release were blocked (**Figures 2D,E**). According to the inhibition of GLP-1 release, insulinotropic actions disappeared (**Figure 2F**). In addition, CuB-mediated insulinotropic actions were required for GLP-1 receptor (**Supplementary Figure 2**). To further examine whether the effects of CuB on plasma GLP-1 release belonged to taste receptors, we performed CuB administration in Gα-gust null mice. There were no significant differences in the blood glucose, plasma GLP-1, and insulin levels with and without CuB administration (**Figures 2G–I**). These results suggest that CuB, which has a potent activator of AMPK, and bitterness, stimulates plasma GLP-1 release by activating AMPK and by stimulating GLP-1 release through Gα-gust. Thus, CuB may cause insulinotropic action.

### CuB Activates Phosphorylation of AMPK and Induces GLP-1 Secretion in Differentiated L-Cells

**FIGURE 3:**
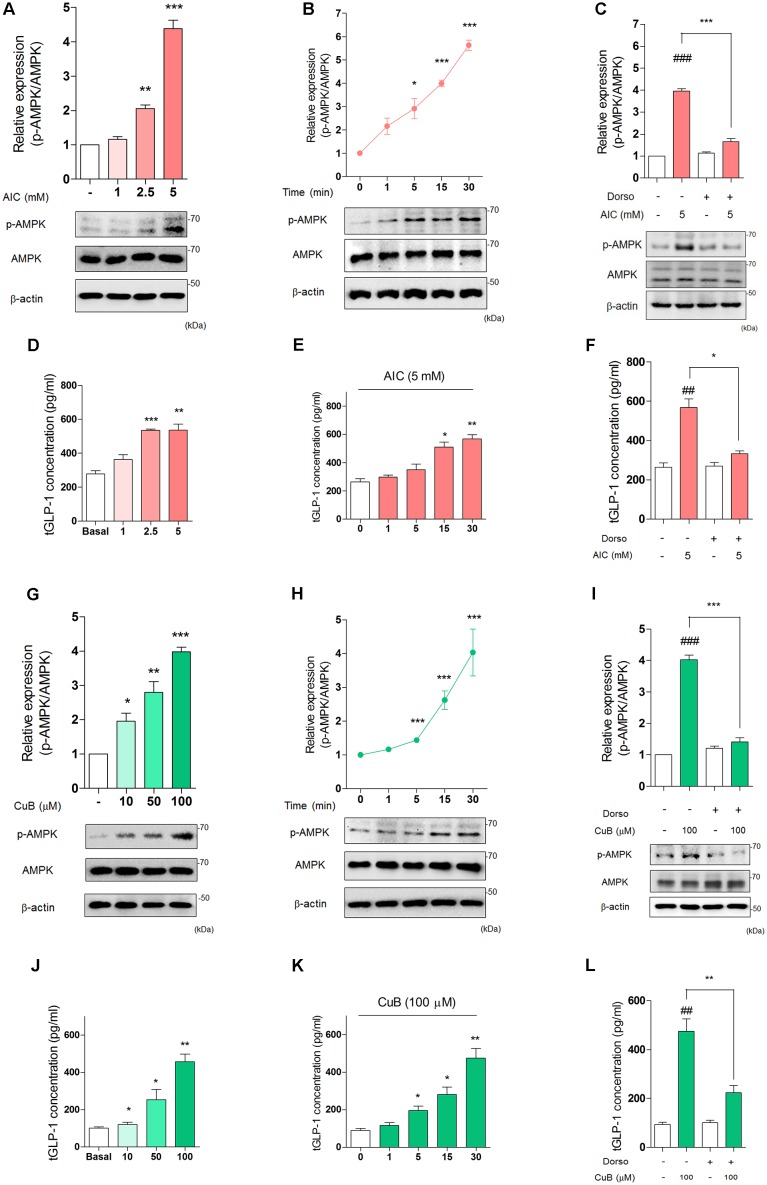
Activation of AMPK stimulates GLP-1 secretion in differentiated NCI-H716 cells. **(A)** AIC treatment dose-dependently induced activation of AMPK in differentiated NCI-H716 cells. **(B)** AIC (5 μμol/l) treatment time-dependently increased activation of AMPK during 30 min. The densitometry of AMPK ratio was represented in the graph. Data are presented as a Mean ± SEM, *n* = 6. ^∗^*P* < 0.05, ^∗∗^*P* < 0.01, ^∗∗∗^*P* < 0.001 vs. control. **(C)** After treatment with Dorso (5 μM) for 30 min, AIC-induced activation of AMPK was inhibited. Data are presented as a Mean ± SEM, *n* = 3. ^###^*P* < 0.001 vs. control, ^∗∗^*P* < 0.05 vs. Dorso + AIC. **(D)** AIC treatment dose-dependently induced GLP-1 secretion from 1 mM to 5 mM in differentiated NCI-H716 for 30 min. **(E)** AIC (5 mM) treatment time-dependently induced GLP-1 secretion for 30 min. Data are presented as a Mean ± SEM, *n* = 8. ^∗^*P* < 0.05, ^∗∗^*P* < 0.01, ^∗∗∗^*P* < 0.001 vs. control. **(F)** After treatment with Dorso, AIC-induced GLP-1 secretion was inhibited. Data are presented as a Mean ± SEM, *n* = 3. ^##^*P* < 0.01 vs. control, ^∗^*P* < 0.05 vs. Dorso + AIC. **(G)** CuB dose-dependently activated AMPK expression levels from 10 μM to 100 μM in differentiated NCI-H716 cells. **(H)** CuB (100 μM) treatment time-dependently increased activation of AMPK during 30 min. The densitometry of AMPK ratio was represented in the graph. Data are presented as a Mean ± SEM, *n* = 6. ^∗^*P* < 0.05, ^∗∗^*P* < 0.01, ^∗∗∗^*P* < 0.001 vs. control. **(I)** After treatment with Dorso for 30 min, CuB-induced activation of AMPK was inhibited. Data are presented as a Mean ± SEM, *n* = 3. ^###^*P* < 0.001 vs. control, ^∗∗∗^*P* < 0.001 vs. Dorso + CuB. **(J)** CuB treatment dose-dependently stimulated GLP-1 secretion from 10 μM to 100 μM in differentiated NCI-H716 for 30 min. **(K)** CuB (100 μM) treatment time-dependently stimulated GLP-1 secretion for 30 min. Data are presented as a Mean ± SEM, *n* = 8. ^∗^*P* < 0.05, ^∗∗^*P* < 0.01, ^∗∗∗^*P* < 0.001 vs. control. **(L)** After treatment with dorsomorphin, CuB-mediated GLP-1 secretion was inhibited. Data are presented as a Mean ± SEM, *n* = 3. ^##^*P* < 0.01 vs. control, ^∗∗^*P* < 0.01 vs. Dorso + CuB. AIC, 5-Aminoimidazole-4-carboxamide-1-β-D-ribofuranoside. Dorso, Dorsomorphin. CuB, Cucurbitacin B.

To establish whether the effects of dorsomorphin on inhibition of GLP-1 release *in vivo* could be mediated through direct actions on the intestinal L-cell, we examined well-defined *in vitro* models of human L-cell, NCI-H716 cell line. We firstly treated AMPK activator, AICAR and observed GLP-1 concentration per protein under differentiated NCI-H716 cells. AICAR treatment increased p-AMPK expression levels in 2.5 mM (Mean: 2.05, SD: 0.1) or 5 mM (Mean: 4.38, SD: 0.24) for 30 min (**Figure 3A**). The p-AMPK expression levels increased time-dependently in 5 mM for 30 min (**Figure 3B**). Dorsomorphin treatment decreased p-AMPK expression levels (**Figure 3C**). Interestingly, AICAR induced GLP-1 secretion in a dose-dependent manner for 30 min (**Figure 3D**) and time-dependent manner in 5 mM (**Figure 3E**). Also, dorsomorphin inhibited AICAR-mediated GLP-1 secretion (**Figure 3F**).

To determine whether CuB activates AMPK in L-cell, we performed a treatment with and without CuB. CuB increased the p-AMPK expression levels at 10 (Mean: 1.95, SD: 0.23), 50 (Mean: 2.79, SD: 0.32), and 100 μM (Mean: 3.98, SD: 0.13) for 30 min (**Figure 3G**). The p-AMPK expression levels increased time-dependently (**Figure 3H**). Dorsomorphin decreased p-AMPK expression levels (**Figure 3I**). Similar to AICAR, CuB induced GLP-1 secretion in a dose-dependent manner (**Figure 3J**) and time-dependent manner (**Figure 3K**). Dorsomorphin inhibited CuB-mediated GLP-1 secretion (**Figure 3L**). These results suggest that AMPK activation induces GLP-1 secretion and may be involved in modulation GLP-1 release in differentiated L-cell.

### CuB-Mediated GLP-1 Secretion and Activation of AMPK Depend on Gα-Gust in Differentiated L-Cells

**FIGURE 4:**
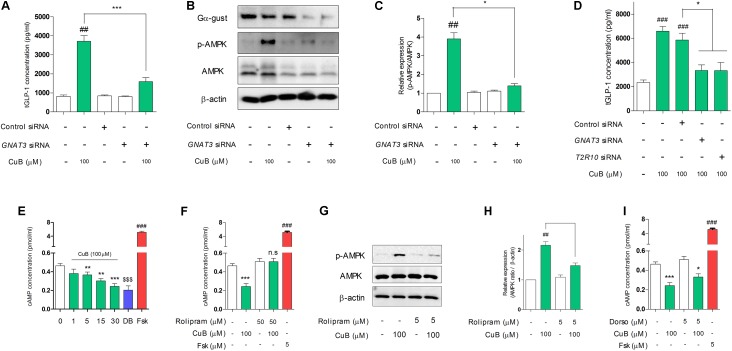
CuB-mediated GLP-1 secretion and activation of AMPK are involved in α-gustducin. **(A)** The siRNAs of *GNAT3* inhibited CuB-mediated GLP-1 secretion in differentiated NCI-H176 cells. Data are presented as a Mean ± SEM, *n* = 5. ^##^*P* < 0.01 vs. control, ^∗∗∗^*P* < 0.001 vs. *GNAT3* siRNA + CuB. **(B)** AMPK expression levels were observed using western blotting under knock down condition of *GNAT3*. **(C)** The knock down of *GNAT3* prevented CuB-mediated activation of AMPK in differentiated NCI-H716. Data are presented as a Mean ± SEM, *n* = 5. ^##^*P* < 0.01 vs. control, ^∗^*P* < 0.05 vs. *GNAT3* siRNA + CuB. **(D)** The transfection of *GNAT3*, *TAS2R10*, and *TAS2R14* siRNA caused to inhibit CuB-mediated GLP-1 secretion in differentiated NCI-H176 cells. Data are presented as a Mean ± SEM, *n* = 5. ^##^*P* < 0.01 vs. control, ^∗∗^*P* < 0.01, ^∗∗∗^*P* < 0.001 vs. Control siRNA. **(E)** CuB treatment led to time-dependently decrease intracellular cAMP. Data are presented as a Mean ± SEM, *n* = 8. ^###^*P* < 0.001 (Frs) vs. control, ^∗∗^*P* < 0.01, ^∗∗∗^*P* < 0.001 (CuB) vs. control, ^$$$^*P* < 0.001 (DB) vs. control. **(F)** CuB-mediated decreasing intracellular cAMP concentration restored by treatment of PDE4D inhibitor (Rolipram, 50 μM). Data are presented as a Mean ± SEM, *n* = 8. ^###^*P* < 0.001 vs. Fsk, ^∗∗∗^*P* < 0.001 (CuB) vs. control. **(G)** AMPK expression levels were observed using western blotting under treatment of rolipram. **(H)** Rolipram treatment prevented CuB-mediated activation of AMPK in differentiated NCI-H716. Data are presented as a Mean ± SEM, *n* = 5. ^##^*P* < 0.01 vs. control, ^∗^*P* < 0.05 vs. Rolipram + CuB. **(I)** CuB-mediated decreasing intracellular cAMP concentration maintained under Dorso treatment. Data are presented as a Mean ± SEM, *n* = 5. ^##^*P* < 0.01 vs. control, ^∗^*P* < 0.05, ^∗∗∗^*P* < 0.001 vs. Dorso + CuB. DB, Denatonium benzoate (10 mM); Fsk, Folskolin (5 μM). Fsk is positive control of activation of cAMP. Dorso, Dorsomorphin. CuB, Cucurbitacin B.

To demonstrate whether the non-response of GLP-1 release by CuB administration in Gα-gust null mice, we examined inhibition study through taste receptor signaling. We firstly performed siRNA transfection of *GNAT3* in differentiated NCI-H716. The knock-down of *GNAT3* attenuated CuB-mediated GLP-1 secretion (**Figure 4A**). Interestingly, CuB-mediated AMPK activation was inhibited by the siRNA *GNAT3* (**Figures 4B,C**). In the systemic inhibition associated with bitter taste receptors such as *GNAT3*, *TAS2R10*, CuB-mediated GLP-1 secretion was inhibited in differentiated NCI-H716 cells (**Figure 4D**). The knock-down efficiency of mRNA is evaluated in **Supplementary Figure 3**. These results suggest that CuB-mediated GLP-1 secretion and AMPK activation are involved in Gα-gust and may result from stimulation of specific bitter taste receptor, TAS2R10.

### CuB-Mediated Intracellular cAMP Reduction Depends on PDE4D in Differentiated L-Cells

To further explore whether CuB-mediated AMPK activation and GLP-1 secretion were involved in Gα-gust-mediated receptor and its signaling pathway, we performed inhibition study of the downstream molecules of the Gα-gust signaling cascade. We firstly tested intracellular cAMP concentration levels. This level decreased in a time-dependent manner in differentiated NCI-H716 after CuB treatment (**Figure 4E**). DB is positive agent of bitterness. Rolipram, PDE4D inhibitor, attenuated the decrease of cAMP (**Figure 4F**). In addition, CuB-mediated AMPK activation was significantly decreased by rolipram treatment (**Figures 4G,H**). However, there was no alteration of intracellular cAMP concentration under dorsomorphin treatment (**Figure 4I**). These results suggest that CuB decreases intracellular cAMP levels dependent on PDE4D and independent on AMPK.

### CuB Stimulates Intracellular Calcium Efflux in Differentiated L-Cells

**FIGURE 5:**
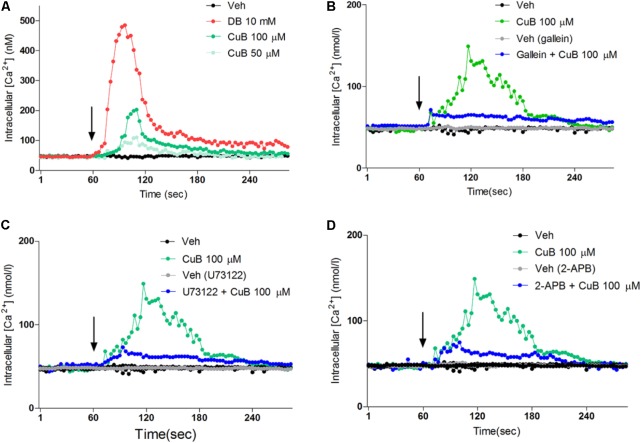
Cucurbitacin B stimulates intracellular calcium through Gβγ-signaling pathway. **(A)** The [Ca^2+^]_i_ increase in response to CuB 100 μM (Green) or, 50 μM (mint) was exhibited. **(B)** After treatment of gallein (10 μM) for 30 min, CuB was treated on each inhibited condition of Gβγ (Blue). CuB-mediated intracellular calcium efflux was inhibited by gallein. **(C)** After treatment of 2-APB (10 μM) for 30 min, the CuB-mediated intracellular calcium efflux was inhibited by 2-APB. **(D)** After treatment of PLCβ, U73122 (10 μM) for 30 min, the CuB-mediated intracellular calcium efflux was inhibited by U73122. Vertical arrow indicates drug application.

To evaluate the intracellular calcium efflux in response to CuB through bitter taste receptor signaling, we undertook single cell imaging to monitor the changes in the concentration of [Ca^2+^]_i_ in differentiated NCI-H716 loaded with a Ca^2+^ indicator dye. In the absence of extracellular calcium, the CuB treatment induced intracellular calcium efflux in differentiated NCI-H716 (**Figure 5A**). To examine whether calcium efflux in response to CuB is involved in Gβγ-signaling, we evaluated the downstream molecules of the Gβγ subunit-signaling cascade, such as Gβγ, PLCβ_2_, and IP_3_R. The calcium efflux in response to CuB was disrupted by gallein, U73122, or 2-APB under differentiated NCI-H176 (**Figures 5B–D**). These results suggest that CuB stimulates intracellular calcium efflux through bitter taste receptor signaling via Gβγ/PLCβ_2_/IP_3_R.

### CuB-Mediated Intracellular Calcium Efflux Activates CAMKKβ/AMPK Signaling in Differentiated L-Cells

**FIGURE 6:**
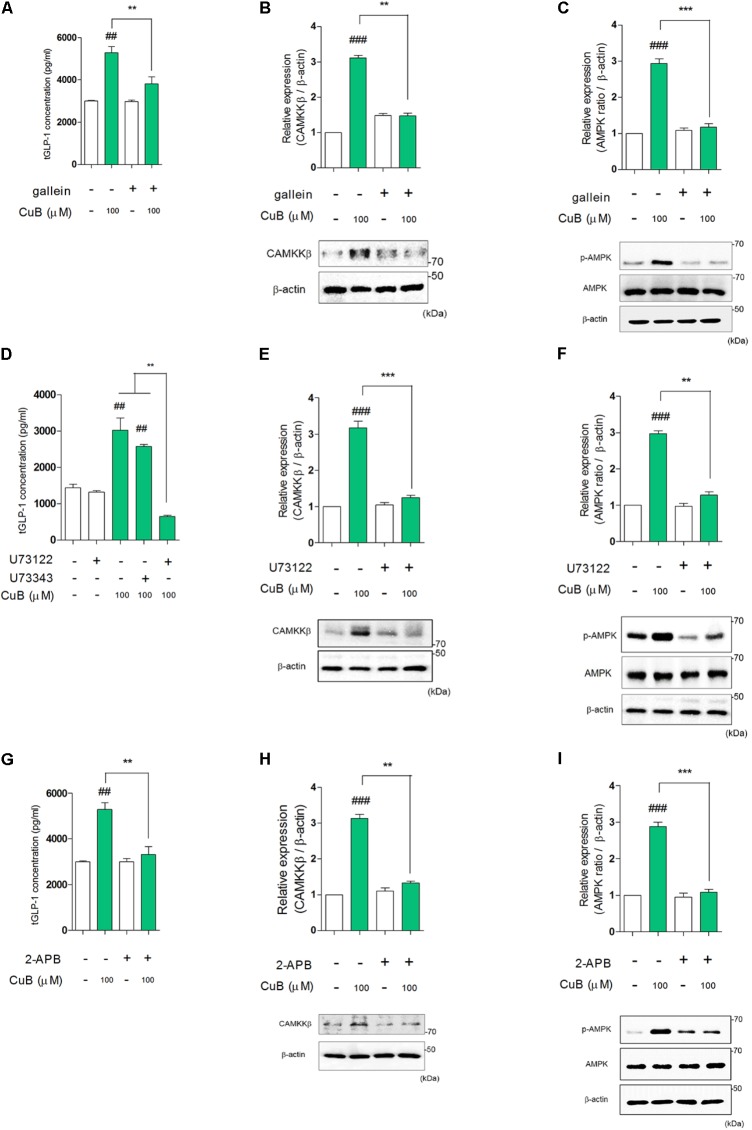
Cucurbitacin B stimulates GLP-1 secretion through Gβγ-signaling pathway. **(A)** CuB-mediated GLP-1 secretion was disturbed under treatment of gallein. Data are presented as a Mean ± SEM, *n* = 5. ^##^*P* < 0.01 vs. control, ^∗∗^*P* < 0.01 vs. Gallein + CuB. **(B,C)** The protein expression levels of CAMKKβ and activation of AMPK were suppressed by Gallein. Data are presented as a Mean ± SEM, *n* = 5. ^###^*P* < 0.01 vs. control, ^∗∗^*P* < 0.01, ^∗∗∗^*P* < 0.001 (CuB) vs. Gallein + CuB. **(D)** CuB-mediated GLP-1 secretion was disturbed under treatment of U73122. Data are presented as a Mean ± SEM, *n* = 5. ^##^*P* < 0.01 vs. control, ^∗∗^*P* < 0.01 (CuB and CuB + U73343) vs. U73122 + CuB. **(E,F)** The protein expression levels of CAMKKβ and activation of AMPK were suppressed by U73122. Data are presented as a Mean ± SEM, *n* = 5. ^###^*P* < 0.01 vs. control, ^∗∗^*P* < 0.01, ^∗∗∗^*P* < 0.001 (CuB) vs. U73122 + CuB. U73343 is inactive analog of U73122. **(G)** CuB-mediated GLP-1 secretion was disturbed under treatment of 2-APB. Data are presented as a Mean ± SEM, *n* = 5. ^##^*P* < 0.01 vs. control, ^∗∗^*P* < 0.01 vs. 2-APB + CuB. **(H,I)** The protein expression levels of CAMKKβ and activation of AMPK were suppressed by 2-APB. Data are presented as a Mean ± SEM, *n* = 5. ^###^*P* < 0.01 vs. control, ^∗∗^*P* < 0.01, ^∗∗∗^*P* < 0.001 (CuB) vs. 2-APB + CuB.

**FIGURE 7:**
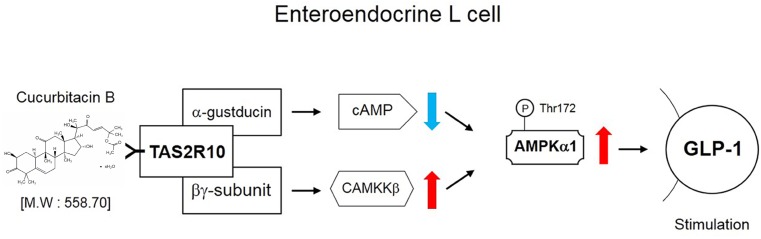
Schematic signaling pathway of CuB effects. The schematic signaling pathway of CuB in differentiated enteroendocrine L-cells.

To examine whether AMPK activation sequentially mediated bitter taste receptor signaling on GLP-1 secretion, we performed to measure GLP-1 concentration under systemic inhibited condition of Gβγ, PLCβ_2_, and IP_3_R under differentiated NCI-H176 cells. Gallein inhibited CuB-mediated GLP-1 secretion (**Figure 6A**). In addition, when down-stream molecules of bitter taste receptor signaling inhibited using PLCβ_2_ and IP_3_R, GLP-1 secretion was disturbed, respectively (**Figures 6D,G**). To determine whether CuB-mediated intracellular calcium efflux was associated with AMPK and its signaling, we investigated CAMKKβ expression levels because CAMKKβ is one of the major up-stream signaling molecules of AMPK. CuB-mediated CAMKKβ and AMPK expression levels were disturbed in response to gallein (**Figures 6B,C**). Consecutively, the CAMKKβ and AMPK expression levels were inhibited under inhibited condition of PLCβ_2_ and IP_3_R using U73122 (**Figures 6E,F**) and 2-APB (**Figures 6H,I**). These results suggest that CuB-mediated intracellular calcium efflux increases CAMKKβ expression, and thus activates p-AMPK. Thus, activation of CAMKKβ/AMPK signaling may be involved in stimulatory effects of GLP-1 via Gβγ/PLCβ_2_/IP_3_R. The CuB action is represented in differentiated enteroendocrine L-cells (**Figure 7**).

## Discussion

The ethnopharmacology using medicinal plants and natural components have developed diverse bioactivities for health care with minimal side effects. If diverse effects of ethnomedicines assimilated with pharmacological evaluation, new natural medicine development will be rapidly applicable research in clinical study by covering phytochemicals-specific mode of action. In this aspect, we tested GLP-1 stimulation in L-cells and agonistic action of taste receptor using CuB. Because our group have previously studied that, the bitter compounds or herbal medicines resulted in Gα-gust-mediated GLP-1 release and alleviated hyperglycemia under the condition of oral glucose tolerance in diabetic mice through activation of taste receptor and of its signaling (Kim et al., 2014a; Suh et al., 2015; Kim and Jang, 2016).

CuB is composed of traditional medicines such as *Begonia heracleifolia, Picrorhiza kurrooa, Echinocystis fabaceae, Wilbrandia ebracteata*, and *Trichosanthes kirilowii* Maximowicz (Chen et al., 2005). Moreover, CuB is one of the derivatives of cucurbitacins including triterpenoid structure, which has significant bioactivity such as anti-inflammatory effects, anti-cancer effects, anti-oxidative stress, and preventive effects against hepatotoxicity (Montesano et al., 2018). With regard to the structural characteristics, CuB is a triterpenoid substance (Nishitoba et al., 1988). Reports on the structural characteristics noted that triterpenoid substances indicated bitterness, and the bitter intensity reflected the spatial distances among three oxygen atoms and the hydrophobic methyl groups, resulting in a crucial role in promoting bitterness in the triterpenoid components (Nishitoba et al., 1988). Meyerhof et al. (2010) also confirmed that the molecular receptive ranges of bitter taste receptors in nature, including CuB and synthetic bitter chemicals, existed in human embryonic kidney (HEK)-293T cells transfected in all 25 human T2Rs. The location of the TAS2R10 agonist binding pocket was structurally identified, and the responsiveness of the agonist-selective position revealed a CuB-binding site on TAS2R10 through mutated residues, including TAS2R_S85V_, TAS2R_Q175L_, and TAS2R_L178F_ (Born et al., 2013). Our results implied that the CuB including the cucurbitane triterpenoid structure had the potential to activate bitter taste receptors, thus stimulate GLP-1 secretion in L-cells and induced plasma GLP-1 release *in vivo*. Because, CuB did not induce plasm GLP-1 release in Gα-gust null mice. Further studies will research the mode-of-action in bitter compounds using transgenic mice, the specific deletion of all bitter taste receptors and will demonstrate the structure-based effect of GLP-1 secretagogue.

In terms of biochemical characteristics, several cucurbitane triterpenoids commonly activate AMPK and AMPK signaling in adipocytes, skeletal myoblast cells, and hepatocytes (Iseli et al., 2013). AMPK played a central role in mediating the appetite-modulating and metabolic effects of many other hormones in the endocrine system (Kola et al., 2006). Metformin, a hypoglycemic agent, activated the p-AMPK, although there was no direct effect on GLP-1 secretion in NCI-H716 cells (Mulherin et al., 2011). Moreover, duodenal mucosal AMPK was activated through the intraduodenal infusion of metformin and contributed to glucose-lowering effects in a model of obesity and diabetes (Duca et al., 2015). However, our group observed different expression of p-AMPK between differentiated and non-differentiated condition. While NCI-H716 cells expressed conserved p-AMPK in non-differentiated condition, this p-AMPK was significantly decreased in differentiated condition. In this condition, our results showed that CuB activated p-AMPK and stimulated dose-dependently and time-dependently GLP-1 secretion in differentiated NCI-H716 cell line.

Moreover, we observed that CuB treatment ameliorated hyperglycemia in *db/db* mice at day 20. The fasting glucose levels were ameliorated in days 10 and 20 in diabetic condition. With reference to given fasting glucose levels in attenuated hyperglycemia, several previous studies using natural compounds also found attenuated hyperglycemia in their experimental results by testing fasting glucose levels of *db/db* mice (Xie et al., 2002; Singh et al., 2012; Jung et al., 2016). This hypoglycemic effect in response to CuB was caused by Gα-gust-mediated GLP-1 release and -dependent AMPK activation. Although the increased expression levels of CAMKKβ and AMPK (T172) in the duodenal intestine did not result from specific L-cells, our results provide an important clue regarding the functional evidence of ameliorated fasting glucose level behind AMPK activation in the intestine of diabetic mice (Harmel et al., 2014; Duca et al., 2015). However, to understand the regulation of fasting glucose levels and the amelioration of hyperglycemic fasting glucose levels, a tremendous amount of specific research related to the regulation of glucose metabolism to understand the relationships between hormones and organ reactions must be done (Jiang and Zhang, 2003).

In this study, we established that both the structural characteristics as an agonist of bitter taste receptors and the biochemical characteristics as an activator of AMPK, stimulated GLP-1 secretion through Gα-gust-mediated bitter taste receptor signaling under differentiated NCI-H716, derived from a poorly differentiated adenocarcinoma of the caecum (de Bruine et al., 1992). In response to bitter compounds, bitter taste receptors are activated for α-subunits such as Gα_i−2_, Gα_i−3_, Gα_14_, Gα_15_, Gα_q_, Gα_s_, and α-transducin as well as βγ-subunits such as Gβ_3_ and Gγ_13_ (Huang et al., 1999; Margolskee, 2002). These subunits then transduce sequent steps in the Gα-gust-PDE or βγ-gustducin-PLC-IP3/DAG pathway (Kim et al., 2014a). Denatonium benzoate (DB) is a representative bitter causing a decrease in intracellular cAMP levels on GLP-1 secretion responding to bitter taste receptors (Kim et al., 2014a). In response to sweet compounds, however, sweet taste receptors are activated in adenylyl cyclase (AC) to cAMP production, which in turn inhibit basolateral K^+^ channels through phosphorylation by the cAMP-activated protein kinase A (PKA) (Margolskee, 2002; Kinnamon, 2012). After that, these subunits transduce sequent steps in the Gα-gust-AC-PKA or βγ-gustducin-PLC-IP_3_/DAG pathway (Kim and Jang, 2015; Kim et al., 2015). According to Lim and Brubaker, Gα_i_ signaling inhibited GLP-1 secretion by reducing intracellular cAMP levels (Lim and Brubaker, 2006). However, DB caused a decrease of Gα_i1,2_ activation, followed by induced GLP-1 secretion (Kim et al., 2014a). Further studies need to demonstrate this opposing regulation of intracellular cAMP in GLP-1 secretion.

In addition, previous studies have suggested that the treatment of glucose and rolipram, an inhibitor of PDE4D, induced a more effective secretion of GLP-1 than the treatment of glucose in the mouse L-cell line GLUTag cells (Ong et al., 2009). This result implied that PDE4D mainly hydrolysed cAMP among several PDEs in L-cells. However, the accumulated cAMP, treated with glucose and rolipram, might lead to the phosphorylation of PKA and maintenance of GLP-1 release through sweet taste receptor signaling rather than having a role in the regulation of GLP-1 release (Jang et al., 2007; Janssen and Depoortere, 2013). Recent studies reported controversial results associated with activation of AMPK on GLP-1 secretion. Firstly, Jiang et al. (2016) suggested that AMPK contributed to the regulation of GLP-1 production and inhibited conditions of AMPK-stimulated GLP-1 secretion in non-differentiated mouse L-cells, STC-1. Secondly, AMPK activators including metformin and AICAR did not increase GLP-1 secretion in L-cells (Mulherin et al., 2011). In addition, Sayers et al. (2016) suggested that the inhibition of AMPK resulted in elevated GLP-1 release in iGluAMPKdKO mice. However, our group earlier needed to discuss the physiological difference between the differentiation and non-differentiation of L-cells, GLP-1 secretagogue effects of dose-dependent response with AICAR, and systemic limitation of iGluAMPKdKO mice as rather controversial results.

From the perspective of physiological difference between the differentiation and non-differentiation of L-cells, although Jiang et al. suggested overexpression of the AMPKα1 gene suppressed GLP-1 secretion in non-differentiated STC-1 cells, our group observed that p-AMPK expression level was conserved in non-differentiated conditions of NCI-H716. Even if it was well-established that both L-cell lines NCI-H716 and STC-1 secreted GLP-1 release in the *in vitro* model, physiological differences of the endocrine differentiation and its mechanisms on GLP-1 secretion were still unclear in the L-cells. In addition, both cell lines derived from human colorectal tumor or murine duodenal secretin tumor cells (Rindi et al., 1990; Reimer et al., 2001). This means that both cell lines may not recapitulate primary L-cells and may have limitations of physiological differences in expression levels and signaling transductions of taste receptors. In aspect of the perspective of GLP-1 secretagogue effects of dose-dependent response with AICAR, although Mulherin et al. (2011) reported that metformin (15–2000 μM) and AICAR (100 and 1000 μM) did not increase GLP-1 secretion in L-cells, Cho and Kieffer (2011) highlighted that metformin acutely increased plasma GLP-1 levels and enhanced the expression of the genes encoding the receptors for both GLP-1 and glucose-dependent insulinotropic polypeptide (GIP). In addition, Kim et al. (2014b) observed that metformin (250 and 500 μM) significantly stimulated GLP-1 secretion in human L-cells. Lastly, although Sayers et al. researched the loss of function of AMPK using mice selectively lacking AMPK in proglucagon-producing cells, this transgenic model deleted AMPK in both pancreatic α cells and intestinal L-cells. The modulation of AMPK activity affected blood glucose levels in pancreatic α cells (Leclerc et al., 2011). Therefore, further study should research interactions between regulation of GLP-1 and AMPK activity in specific deletion models of AMPK. In addition, studies examining the physiological regulations between AMPK and gut hormones including GLP-1 should resolve gene expression associated with taste receptors in human and mouse primary L-cells.

We showed that the loss of Gα-gust affects the activation of AMPK in L-cells. Thus, rather than a global impact on GLP-1 release in the gut, Gα-gust seems to regulate not only the activation of AMPK but also α- and βγ-subunit-signaling transduction on GLP-1 secretion. In the absence of Gα-gust, behavioral and electrophysiological responses were inhibited in response to sweet and bitter tastants (Wong et al., 1996). The Gα-gust impacted on food intake and gastric emptying by regulation of ghrelin (Janssen et al., 2011). The ghrelin stimulated hypothalamic AMPK, which was involved in the regulation of orexigenic neuropeptides, neuropeptide Y (NPY), and anorexigenic neuropeptide pro-opiomelanocortin-α (POMC) (Andersson et al., 2004; Kola et al., 2005). The anorectic signal, in response to GLP-1, played a part in inhibiting hypothalamic AMPK, causing enhanced acetyl-CoA carboxylase (ACC). Taken together, these findings implied that Gα-gust, whose expression can be stimulated by sweet or bitter tastants, might be involved in a complex signaling network involving NPY, POMC, GLP-1, and AMPK that affects the regulation of appetite and feeding behavior.

## Author Contributions

K-HK generally performed all experiments of *in vivo* and *in vitro* including management of Gα-gust null mice and wrote manuscripts. I-SL and JP partially contributed to blood isolation on Gα-gust null mice and *db/db* mice. E-JA and YK contributed to WB. H-JJ conducted academic plan of all experiments.

## Conflict of Interest Statement

The authors declare that the research was conducted in the absence of any commercial or financial relationships that could be construed as a potential conflict of interest.

## Abbreviations

AICAR: aminoimidazole-4-carboxamide-1-β-D-ribofuranoside
AMPK: AMP-activated protein kinase
CAMKKβ: calcium/calmodulin-dependent protein kinase kinase β
CuB: cucurbitacin B
DAG: diacylglycerol
DB: denatonium benzoate
Ex9: exendin 9-39
GLP-1: glucagon-like peptide-1
GLP-1R: glucagon-like peptide-1 receptor
GPCR: G-protein-coupled receptors
Gα-gust: G-protein α subunit of gustducin
HbA1c: hemoglobin A1c
IP_3_: inositol 1,4,5-triphosphate
L-cells: enteroendocrine L cells
PDED: phosphodiesterase D
PIP_2_: phosphatidylinositol 4,5-bisphosphate
PKC: protein kinase C
PLCβ_2_: phospholipase β_2_
PYY: peptide YY
T2DM: type 2 diabetes mellitus
TRPM5: transient receptor potential channel 5.
