# Supplementary figures and images for: Cucurbitacin B Induces Hypoglycemic Effect in Diabetic Mice by Regulation of AMP-Activated Protein Kinase Alpha and Glucagon-Like Peptide-1 via Bitter Taste Receptor Signaling
Source: Front Pharmacol. 2018 Sep 21;9:1071. doi: 10.3389/fphar.2018.01071 (PMC6161541; doi:10.3389/fphar.2018.01071)

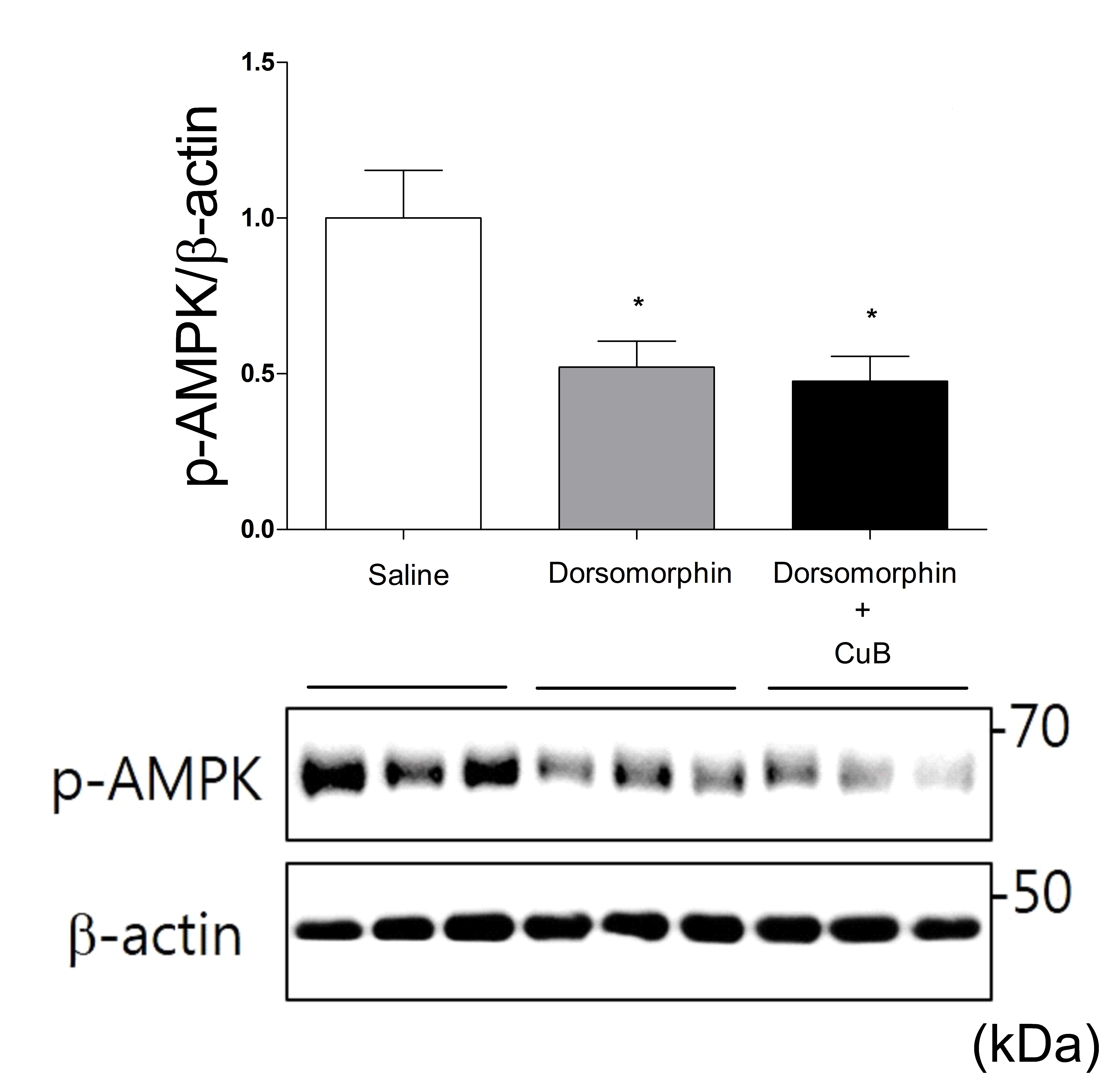

Supplement: Supplementary file 1 [file Image_1.JPEG]

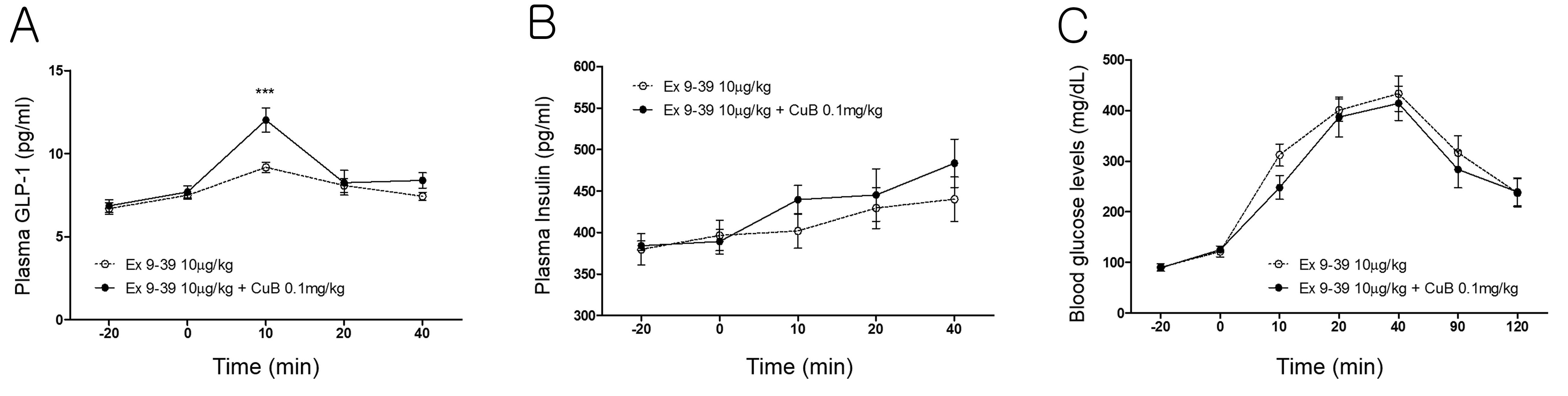

Supplement: Supplementary file 2 [file Image_2.TIF]

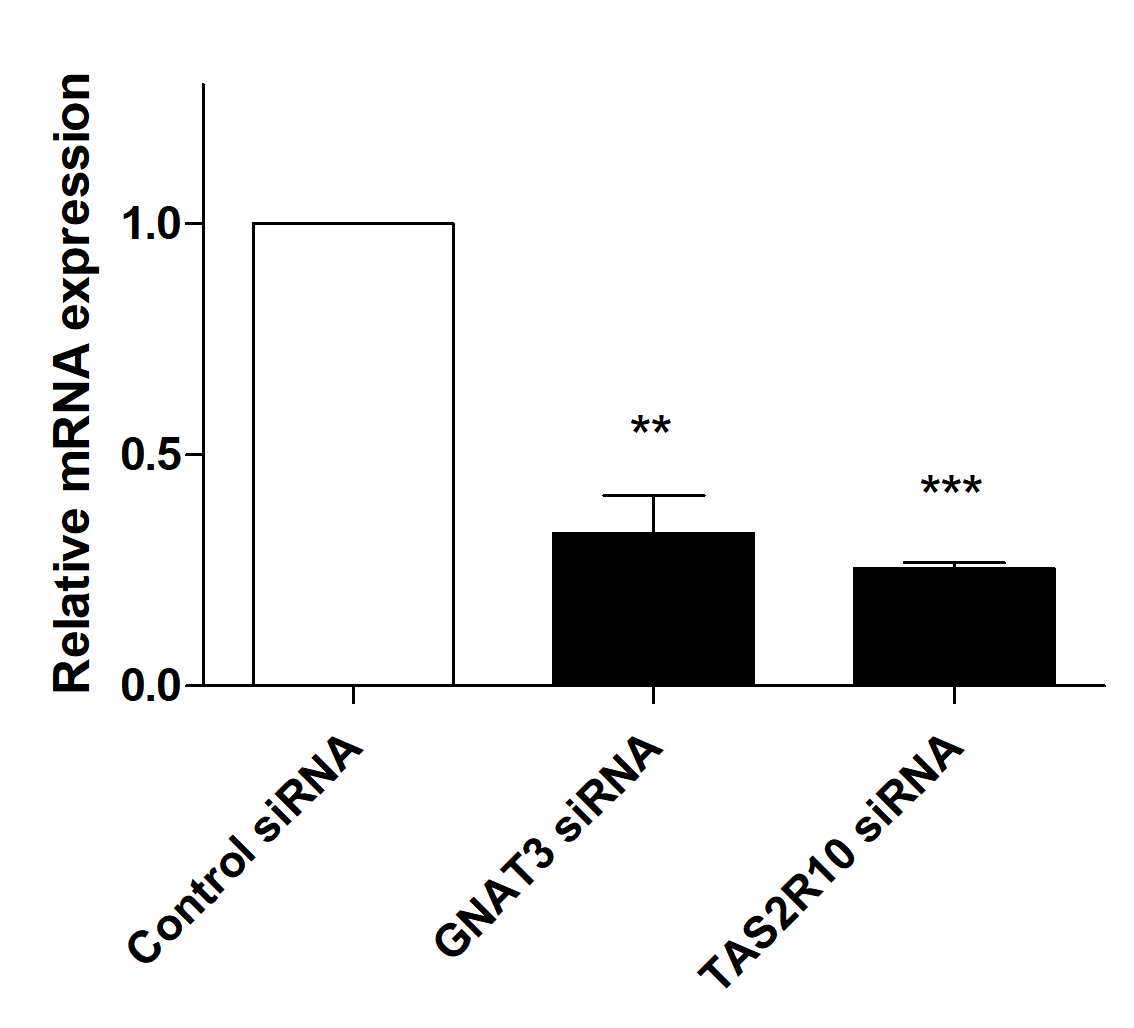

Supplement: Supplementary file 3 [file Image_3.TIF]
