# Supplementary material for: Cucurbitacin B Induces Hypoglycemic Effect in Diabetic Mice by Regulation of AMP-Activated Protein Kinase Alpha and Glucagon-Like Peptide-1 via Bitter Taste Receptor Signaling
Source: Front Pharmacol. 2018 Sep 21;9:1071. doi: 10.3389/fphar.2018.01071 (PMC6161541; doi:10.3389/fphar.2018.01071)
Supplement: Supplementary file 4 [file Table_1.DOCX]

Supplementary figure.

Supplementary figure 1. After i.v injection (2mg/kg) of dorsomorphin, the expression levels of p-AMPK were decreased in the skeletal muscle. Data are presented as a Mean ± SEM, *n*=8. **P*<0.05 vs. Saline.

Supplementary figure 2. (A) Fasted mice were injected through i.p with Ex 9 (10 μg/100 μl per mice), with (black square) or without (black square) 0.1mg/kg CuB before OGTT, and their blood glucose were measured using Acu-Check performa in end of tail. (B & C) After OGTT, plasma GLP-1 and plasma insulin were measured using multiplex system. Data are presented as a Mean ± SEM, n=8 of the C57bL/6 group treated with or without CuB. **P < 0.01, ***P < 0.001 vs. Ex 9. Ex 9, exendin9-39.

Supplementary figure 3. The siRNA transfection transfection represented down-regulated mRNA expression on differentiated NCI-H716 cells. The mRNA expression levels of *GNAT3* and *TAS2R10* were significantly decreased. Data are presented as a Mean ± SEM, *n*=8, ***P*<0.01 vs. Control siRNA.
